# Human Exposure to *Anaplasma phagocytophilum* in Two Cities of Northwestern Morocco

**DOI:** 10.1371/journal.pone.0160880

**Published:** 2016-08-17

**Authors:** Sarah Elhamiani Khatat, Hamid Sahibi, Mony Hing, Ismail Alaoui Moustain, Hamid El Amri, Mohammed Benajiba, Malika Kachani, Luc Duchateau, Sylvie Daminet

**Affiliations:** 1 Department of Pathology and Veterinary Public Heath, Institut Agronomique et Vétérinaire Hassan II, Rabat, Morocco; 2 Department of Companion Animals, Faculty of Veterinary Medicine, Ghent University, Merelbeke, Belgium; 3 National Reference Laboratory for *Anaplasma phagocytophilum*, Laboratory of Clinical Biology, Queen Astrid Military Hospital, Brussels, Belgium; 4 Central Health Services of the Royal Gendarmery, Rabat, Morocco; 5 Laboratory of the Royal Gendarmery, Rabat, Morocco; 6 Regional Transfusion Center, Rabat, Morocco; 7 College of Veterinary Medicine, Western University of Health Sciences, Pomona, California, United States of America; 8 Department of Comparative Physiology and Biometrics, Faculty of Veterinary Medicine, Ghent University, Merelbeke, Belgium; University of Maryland, College Park, UNITED STATES

## Abstract

*Anaplasma phagocytophilum* is an emerging tick-borne zoonosis with extensive increased interest. Epidemiological data are available in several regions of the USA, Europe and Asia in contrast to other parts of the world such as North Africa. Blood samples of 261 healthy individuals divided in two groups i.e., dog handlers and blood donors were analysed. Indirect immunofluorescent assay using a commercial kit was performed to detect specific *A*. *phagocytophilum* IgG. Two dilutions were used to assess the prevalence of seroreactive samples. Demographic variables were assessed as potential risk factors using exact logistic regression. Seropositivity rates reached 37% and 27% in dog handlers and 36% and 22% in blood donors. No statistically significant differences were found in the prevalence rates between the two groups. Analysis of risk factors such as gender, age groups, outdoor activities, self-reported previous exposure to ticks, or contact with domestic animals (dogs, cats, ruminants and horses) did not shown any significant difference. *A*. *phagocytophilum* exposure was common in both high-risk population and blood donors in Morocco.

## Introduction

*Anaplasma phagocytophilum* is an obligate intracellular gram negative bacterium that infects neutrophils. The bacterium causes an emerging zoonotic tick-borne disease (TBD) called granulocytic anaplasmosis [1]. *A*. *phagocytophilum* is mostly transmitted to humans through the bites of ticks of the *Ixodes* genus. However, other modes of transmission have been described including transplacental transmission, percutaneous exposure or inhalation of the contaminated blood of deer, nosocomial infection following direct contact with blood and respiratory secretions and through blood transfusions [2, 3].

Human granulocytic anaplasmosis (HGA) is an unspecific flu-like illness that is typically characterized by the acute onset of fever, headache, chills, myalgia, malaise, nausea, and cough. Depending on several risk factors, which include advanced age, immunosuppression, co-morbidities and delays in the onset of treatment, HGA can be mild or fatal [4, 5]. Life-threatening complications occur in 3% of cases. Consequently, half of the HGA cases are hospitalized and up to 17% of patients require admission to intensive care units, especially when the diagnosis and treatment are delayed [1, 4]. Therefore, the Infectious Diseases Society of America recommends that antimicrobial therapy be given to every person suspected of having HGA on the basis of their clinical presentation, so as not to delay the treatment [6]. Due to the potentially serious outcome and the difficulty of the diagnosis, epidemiological data on the prevalence and distribution of human cases within a country are important to increase awareness of physicians and to develop adapted public health strategies to prevent and control this disease [7].

HGA commonly occurs in the USA and Europe, and it is increasingly diagnosed in some Asian countries [6, 8]. In the USA, at least 15,952 HGA cases were reported since 1995 and a 12-fold increased incidence has been observed between 2001 and 2011 [4]. In China, the exposure to *A*. *phagocytophilum* has continuously increased from 8.8% to 59.2% in high-risk populations between 2006 and 2009 [3]. Despite a moderate to high seroprevalence in several countries, HGA is still unrecognized and rarely diagnosed due to several factors including limited epidemiological information, difficult diagnosis, asymptomatic or subclinical infections and the lack of awareness among physicians and the public [2, 3, 6]. Moreover, the occurrence of HGA is unknown in many regions of the world such as Oceania, South America, Africa, and in large regions of Asia. To the author’s knowledge, no data are available in North Africa on either the occurrence of HGA or the prevalence of human exposure to *A*. *phagocytophilum*. However, ticks are abundant in this region and might represent a hazard for both animal and human public health [9]. Therefore, we carried out a cross-sectional epidemiological serologic survey to investigate the potential human exposure to *A*. *phagocytophilum* in Morocco.

## Materials and Methods

### Study population

Between June and September 2015, 261 healthy individuals from two groups were sampled from three cities of Morocco. The first group included 144 military and police dog handlers from the first kenel of the Royal Forces Army of Benslimane and the kenel of the Royal Gendarmerie of Temara. This group was considered to be at a high risk for TBDs because of their regular contact with dogs and outdoor occupational activities. The data collected on this group included age and exposure to ticks. The second group included 117 blood donors from the Regional Transfusion Centre of Rabat. All of the blood donors were informed on the purpose of the survey and signed informed consent forms before enrollment. An epidemiological report was completed for each blood donor containing data on the age, city of residence, occupation, travels outside Morocco during the previous year, outdoor activities, tick exposure and potential contact with dogs and other domestic animals (i.e., cats, horses and ruminants). The study protocol was approved by the Ethical Committee for Biomedical Research of the Mohammed V University of Rabat (n°698; July 10, 2014) and the Ministry of Health of Morocco (n°965; June 12, 2014).

### Blood sampling

For each person included in the study, 5 ml of non-anticoagulated blood was collected from the elbow groove veins. Blood samples were centrifuged at 3500 rpm during 10 min at 15°C and sera were aliquoted and stored at -32°C until shipment to the National Reference Laboratory for *A*. *phagocytophilum* in Belgium.

### Serological tests

**Fig 1:**
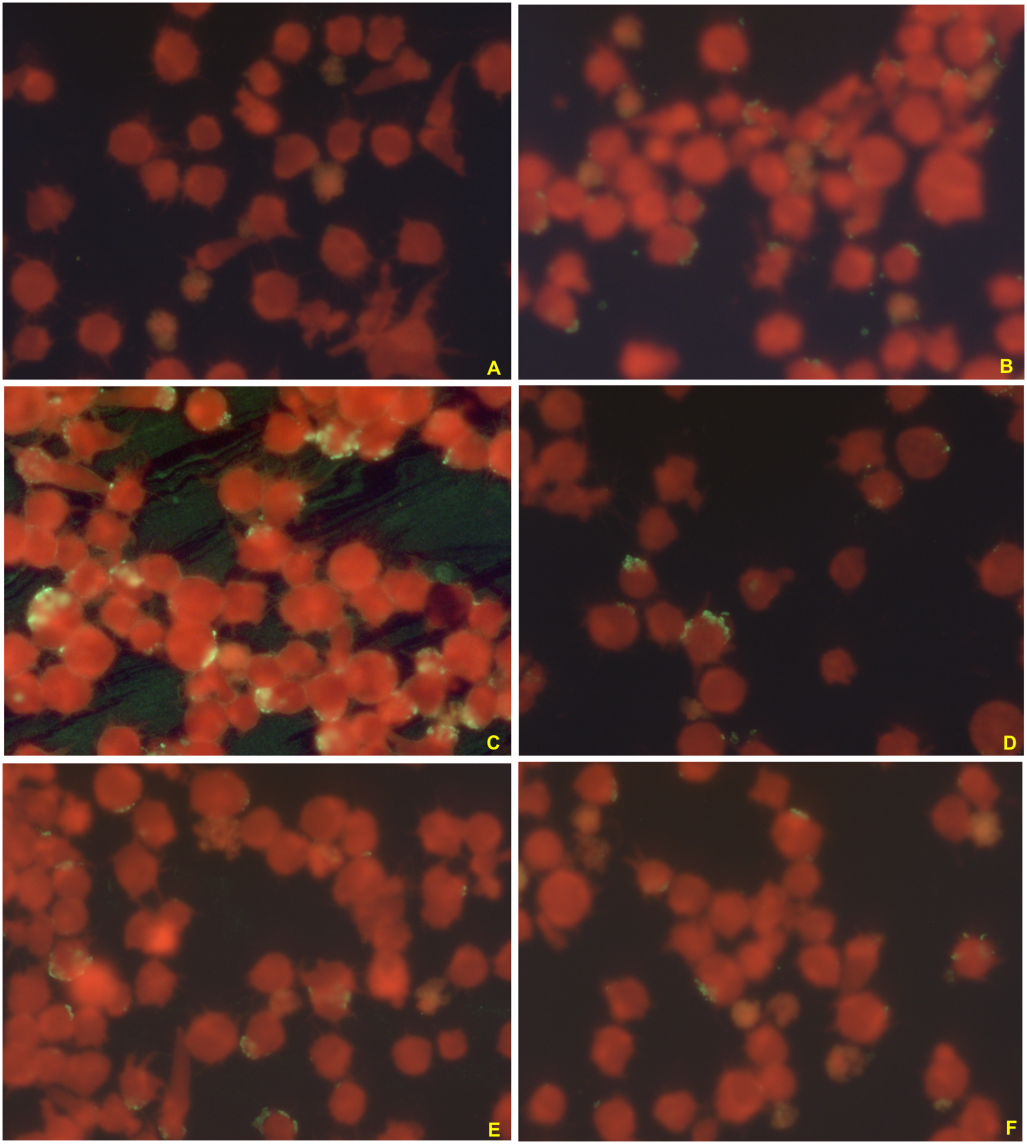
Photographs of ultraviolet light microscopy (×400) of *A*. *phagocytophilum* IgG semi-quatitative IFA measurement using a commercial kit (Focus Diagnostics, Cypress, California, USA) showing a negative control (A), a positive control (B) and for positive dilutions i.e., 1:64 (C), 1:128 (D); 1:256 (E) and 1:516 (F) from the same patient. The positivity is set on the observation of green morulae surrounding the cell’s cytoplasmic membrane.

Immunoglobuline G (IgG) antibodies were detected against *A*. *phagocytophilum* by a semi-quantitative indirect immunofluorescent assay (IFA) using a commercial kit (Focus Diagnostics, Cypress, California, USA) containing HL60 cells infected with a human isolate of *A*. *phagocytophilum* HGE-1 according to the manufacturer’s instructions. Briefly, 5 μL of serum were diluted in 315 μL of phosphate-buffer saline (PBS) (0.01 M, pH = 7.2±0.1). The positive IgG control was also diluted into the following five dilutions: 1:2, 1:4, 1:8, 1:16 and 1:32. Then, 25 μL of diluted sera were added in the wells of each slide. The first line of the first slide contained the negative IgG control and the five dilutions of the positive IgG control. The slides were incubated in humid chambers between 35 and 36.5°C for 30 min, then they were washed with the PBS solution followed by distilled water to eliminated non conjugated serum antibodies. Next, 25 μL of the conjugate containing human IgG and fluorescein were added to each well. The slides were incubated again and washed as described above. Finally, the slides were dried and coverslipped using a mounting medium and were examined under ultraviolet light microscopy (×400). The titer was defined as the reciprocal of the highest dilutions of serum with the homogeneously stained cytoplasmic morulae (Fig 1). A serum titer of ≥ 1:64 was considered as positive for *A*. *phagocytophilum* IgG according to the instructions provided by the manufacturer. Samples that were positive at the first dilution of 1:64 were then further diluted to 1:128 and those remaining positive at the second dilution were then titered at 1:256 and 1:512. Ten samples were reassessed by a blinded technician from the laboratory at a dilution of 1:64 and the results were confirmed in all cases.

### Statistical analyse

A statistical analysis was performed using SAS version 6.4 (SAS Institute Inc., Car, NC, USA). The exact logistic regression model was fitted to compare seroreactivity rates between both dog handler and blood donor groups and between gender, the presence or absence of outdoor activities, exposure to ticks, dogs or other domestic animals inside the blood donor group. The statistical significance was set at 5%. The results were summarized in terms of the odds ratio with a 95% confidence interval.

## Results

Eight samples were excluded due to hemolysis that could interfere with the results according to the manufacturer instructions. A total of 138 dog handlers (54.5%) and 115 blood donors (45.4%) were included in the study. The majority of blood donors (105/115) lived in Rabat or the surrounding cities whereas nine blood donors were from other cities (Table 1).

**Table 1:** Distribution of the number of blood donors and of positive samples for both dilutions according to city.

Administrative region	City	Distance to Rabat (km)	Number of blood donors	Number of positive IgG 1: 64	Number of positive IgG 1: 128
**Rabat-Salé-Kénitra**	Rabat		55	19	10
	Salé	5.2	32	11	6
	Temara	8.0	13	5	2
	Ain El Aouda	30.0	1	1	1
	Sidi Allal El Bahraoui	37.7	1	0	0
	Kenitra	55.0	1	0	0
**Casablanca-Settat**	Bouznika	39.6	1	1	1
	Benslimane	60.0	1	0	0
**Béni Mellal-Khénifra**	Khenifra	237.4	1	1	1
	Beni Mellal	233.1	1	1	1
**Tanger-Tétouan-Al Hoceima**	Tangier	250.0	1	1	1
**Souss-Massa-Drâa**	Tinghir	477.0	1	0	0
	Agadir	547.0	3	1	1
	Tizi n'Tichka		1	0	0
**Guelmim-Oued Noun**	Sidi Ifni	686.0	1	0	0

Abbreviations: IgG, immunoglobuline G.

The city of origin was unavailable for one blood donor. All dog handlers were men between 21 and 51 years of age (average age: 33 years). The blood donors group included 63 men (54.8%) and 52 women (42.2%), and their ages ranged from 18 to 61 years (average age: 39 years). The distribution according to the epidemiological variables in the two groups is summarized in Table 2.

**Table 2:** Distribution of age, sex, exposure to ticks, contact with dogs or other domestic animals and travel history outside Morocco in both dog handlers (n = 138) and blood donors (n = 115) groups.

Variables		Dog handlers (%)	Blood donors (%)
**Sex**	Men	138 (100)	63 (54.8)
	Women	0 (0.0)	52 (45.2)
**Age** (years-old)	≤20	1 (0.7)	7 (6.1)
	21–30	78 (56.5)	26 (22.6)
	31–40	47 (34.1)	27 (23.5)
	41–50	10 (7.2)	36 (31.3)
	>50	1 (0.7)	19 (16.5)
**Exposure to ticks**		2 (1.4)	7 (6.1)
**Outdoor activities**		138 (100)	86 (74.8)
**Contact with dogs**		138 (100)	11 (9.6)
**Contact with other domestic animals**		-	17 (14.8)
**Travel**		0 (0.0)	18 (15.7)

Outdoor activities in the forest or rural areas were either occupational or for leisure (picnic, hiking, jogging, walking or hunting). More than half of the blood donors (47/86) reported their outdoor activities in the region of Rabat-Salé-Kenitra région and 20.9% (18/86) reported their activities in other Moroccan regions including Moulay Bousselham, Gharb region, Tangier, Ouazzane, Rif mountain, Ifrane, Azrou, Khouribga, Oued Zem, Nador, Taza, Oujda, El Jadida, Safi, Essaouira, Agadir, Tiznit, Dakkhla, Beni Mellal, Azilal, Marrakech, Ait Baha, Zagora, Taroudant and the High Atlas mountains. The remaining 21 blood donors (24.4%) reported their outdoor activities in both Rabat-Salé-Kenitra and other regions. Previous exposure to ticks was recorded in 1.4 (2/138) and 6.1% (7/117) of dog handlers and blood donors, respectively. Travel outside of Morocco was recorded in 15.6% (18/115) of blood donors and ten traveled to two or more countries.

**Fig 2:**
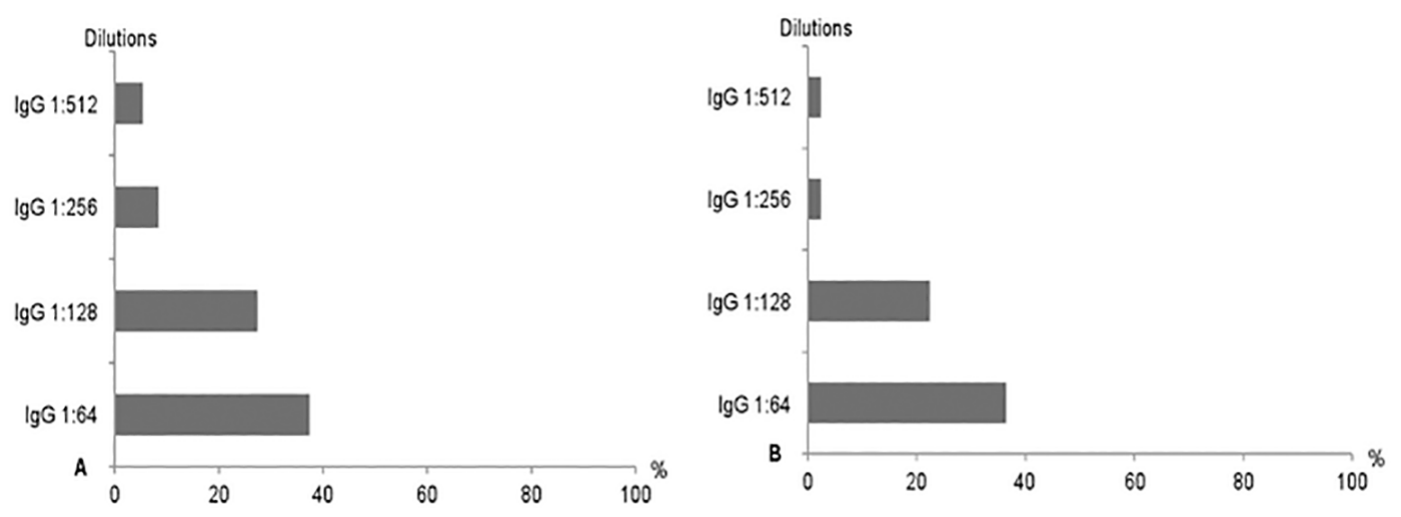
Distribution of positivity rates for the four *A*. *phagocytophilum* IgG dilutions (i.e, 1:64, 1:128, 1:256 and 1:516) in both dog handlers (A) and blood donors (B) groups.

**Fig 3:**
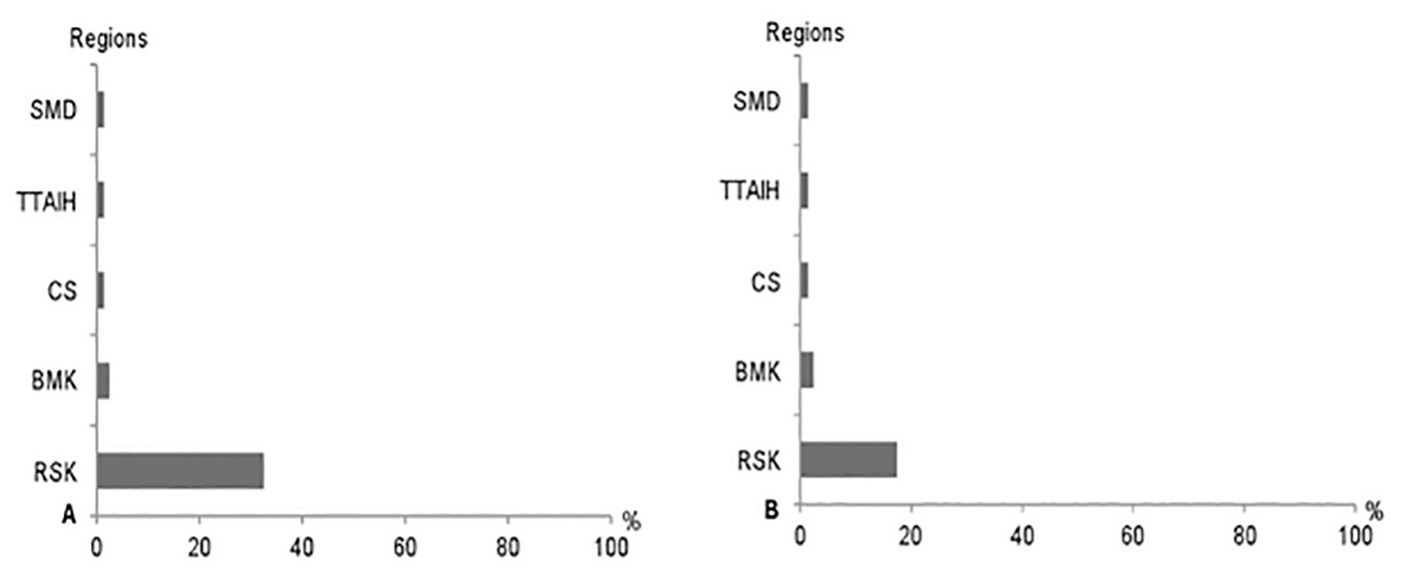
Distribution of *A*. *phagocytophilum* IgG positivity rates in blood donors according to the region of living the Rabat-Salé-Kénitra (RSK), Casablanca-Settat (CS), Tangier-Tétouane-Al Hoceima (TTAlH) and Souss-Massa-Drâa (SMD) regions and for both 1:64 (A) and 1:128 (B) dilutions.

The seropositivity rates for *A*. *phagocytophilum* IgG at the first dilution reached 37.0% (51/138) and 35.7% (41/115) in dog handlers and blood donors, respectively (Table 3 and Fig 2). At the second dilution, 27.5% (38/138) and 21.7% (25/115) of sera were still reactive in the dog handlers and the blood donors groups, respectively (Table 3 and Fig 2). Most seropositive blood donors for both dilutions (i.e., 1:64 and 1:128) were from the region of Rabat-Salé-Kénitra (Fig 3).

**Table 3:** Number of seropositive samples in both dog handlers (n = 138) and blood donors (n = 115) groups at the four different dilution.

Variables	Dog handlers (%)	Blood donors (%)	OR	95%CI	P value
**IgG 1:64**	51 (37.0)	41 (35.7)	1.05	0.61–1.83	0.90
**IgG 1:128**	38 (27.5)	25 (21.7)	1.37	0.74–2.56	0.31
**IgG 1:256**	11 (8.0)	2 (1.7)	-	-	-
**IgG 1:512**	7 (5.1)	2 (1.7)	-	-	-

Abbreviations: IgG, immunoglobuline G; OR, odds ratio; 95%CI, 95% confidence interval.

No statistically significant differences were found between the two groups considering the seroreactivity rates at both dilutions (Table 3). Similarly, no statistically significant differences were found in the blood donor group when comparing between gender, age groups, the presence of outdoor activities, exposure to ticks, and contact with dogs or other domestic animals at both dilutions (Table 4). In the dog handlers group, 11 (8.0%) and 7 (5.1%) of the sera were still positive when further diluted to 1:256 and 1:512, respectively (Table 3 and Fig 2). Only two of the samples remained positive at both 1:256 and 1:512 in the blood donors group (Table 3 and Fig 2).

**Table 4:** Distribution of seropositive samples according to the gender, age group, presence of outdoor activities, contact with ticks, dogs or other animals and travel history in the blood donor group for both 1:64 (n = 41) and 1:128 (n = 25) dilutions.

Varaiables		IgG 1:64				IgG 1:128			
		Number (%)	OR	95%CI	P value	Number (%)	OR	95%CI	P value
**Gender**	Men	23 (56.1)	1.08	0.47–2.52	0.85	16 (64.0)	1.62	0.60–4.62	0.37
	Women	18 (43.9)				9 (36.0)			
**Age** (years-old)	≤20	2 (4.9)	1.03	0.99–1.06	0.12	2 (8.0)	1.00	0.96–1.03	0.85
	21–30	9 (22.0)				7 (28.0)			
	31–40	6 (14.6)				6 (24.0)			
	41–50	14 (34.1)				3 (12.0)			
	>50	10 (24.4)				7 (28.0)			
**Exposure to ticks**		2 (4.9)	1.41	0.22–15.45	1.00	1 (4.0)	1.71	0.19–82.09	1.00
**Outdoor activities**		30 (73.2)	1.44	0.56–3.63	0.51	17 (68.0)	1.73	0.59–4.89	0.31
**Contact with dogs**		5 (12.2)	0.64	0.15–2.84	0.52	2 (12.0)	0.72	0.15–4.55	0.70
**Contact with other domestic animals**		4 (9.8)	1.96	0.55–8.87	0.41	2 (8.0)	2.29	0.47–22.08	0.36
**Travel**		7 (17.1)	-	-	-	5 (20.0)	-	-	-

Abbreviations: IgG, immunoglobuline G; OR, odds ratio; 95%CI, 95% confidence interval.

## Discussion

To the author’ s knowledge, this is the first report investigating human exposure to *A*. *phagocytophilum* in Africa. In Europe, the USA and Asia, several reports have investigated the prevalence of human exposure in blood donors [10–19] and in high-risk populations including people living in forest areas and forestry workers [10, 12, 16, 20–25], people living in rural areas and farmers [3, 8, 19, 26–28], hunters [8, 11], national parks rangers [29–30], military personnel [31], people in close contact with domestic animals [7, 21], and people at high risk of exposure or previously exposed to ticks [7, 32, 33]. The prevalences recorded in high-risk populations or in endemic areas were up to 32%, 35.6%, and 33.7% in Europe [23], the USA [34], and China [35], respectively. However, several serological methods and cutoffs were used, which made the comparison between these studies difficult [31]. When comparing the results of military dog handlers obtained in this study at the threshold of 1:64, i.e., 37%, with other Chinese [24, 35] and European [12, 21, 23, 25, 28, 32, 36] reports using the same method and the same cutoff, it appears that the prevalence in Morocco is higher. The highest prevalences recorded in both China and Europe were 20% [7] and 9.6% [25], repectively. One study from Cyprus reported a prevalence of 32% with a cutoff of 1:128 [23] which is slightly higher than the results found in Morocco at the same cut off (27.5%). When comparing the results of this study with high-risk populations from other European Mediterranean countries such as Italy (8.8%) [22], Portugal (5.4%) [17], and Spain (1.4%) [21], the prevalence found in Moroccan dog handlers is higher at both the first and second dilutions. Moreover, the prevalence in Moroccan dog handlers is even higher than the prevalence found in patients with clinical signs and history of tick bites in Belgium [5].

Because high-risk populations were shown to have a significantly higher prevalence of *A*. *phagocytophilum* exposure [3,16, 31] they may not reflect the true exposure of the general population of the same country [7]. Therefore, a more representative sample including blood donors with more diverse social and intellectual levels, occupational and leisure activities would be a better sample to estimate the prevalence of *A*. *phagocytophilum* exposure in Morocco. In addition, the high seroprevalence rates in blood donors of some geographic locations, the potential asymptomatic or subclinical evolution of the disease, the survival of *A*. *phagocytophilum* in refrigerated blood products and documented transfusion-transmitted HGA cases, provide further reasons to screen blood donors in Morocco. Although only a few cases of transfusion-transmitted anaplasmosis have been reported, *A*. *phagocytophilum* infection is among the TBDs that are considered to represent a potential risk for transmission by blood transfusion. In addition, because sharing blood products between different areas is growing, such an acute illness after blood transfusion should be included in the differential diagnosis even in nonendemic areas [2, 37]. Our results showed that even in the blood donor group, high prevalences of 35.7% and 21.7% at both the 1:64 and 1:128 dilutions, respectively, were recorded. When compared to European prevalences in blood donors using the same method and the same cutoffs, these results are higher than those published in Poland (2%) [12], and Austria (9%) [15], but they are similar to those from Greece (21.4%) [18]. Without taking into account the method and the cutoffs, the results from Moroccan blood donors are even higher than those from several US [13, 14] and European reports [10, 11, 16, 17, 19, 33, 38]. In several reports that compared the seroreactivity rates of blood donors to those of high-risk populations, significant differences were found [10,16, 17]; these findings are in contrast to our report. These differences could be due to the relatively high proportion of the blood donors that report outdoor activities, which could then increase the possible exposure to ticks and thus predispose them to *A*. *phagocytophilum* infection.

No risk factors were identified in this survey in either group. Similarly, some reports failed to identify specific demographic variables as potential risk factors [12, 17, 23, 25, 28]. In contrast, other reports demonstrated that seropositivity rates were significantly higher in men [3, 23, 30], in age groups from 20 to 40 [3, 23] and 40 to 65 years of age [32] and the rates increased with age [29]. Seropositivity rates among Moroccan blood donors were higher in men especially for the dilution of 1:128 and lower in the age group ≤20 year-old, although this was not statistically significant. No statistically significant associations between seroreactivity rates and the contact with animals or outdoor activities were found in this study. However, the chance of coming into contact with infected ticks depends on several epidemiological and ecological factors, such as the environment, the presence of appropriate hosts and reservoirs. Consequently, outdoor activities that are especially related to wooded areas, meadow habitats and grasslands are considered to be some of the major risk factors for acquiring TBDs [39]. Moreover, a large number of participants to a study from Germany mentioned contracting their most recent tick bite in their gardens and half of the participants with past exposure to *A*. *phagocytophilum* listed gardening as a regular leisure activity; despite a comparatively low risk of exposure associated with this activity. Therefore, public health measures to increase awareness for TBDs should also target the large portion of the population who are involved in comparatively low risk outdoor activities such as gardening, cycling or walking [32]. Although not statistically significant, a high proportion (74.8%) of the blood donors mentioned participating in outdoor activities. Consequently, the obvious popularity of outdoor activities may predispose a large number of people to the risk of infection by *A*. *phagocytophilum*. Only a small portion (3.6%) of the tested population had a history of tick exposure without any significant difference between both groups. Similarly, several surveys did not find any association between self-reported exposure to ticks and the seroreactivity rates of *A*. *phagocytophilum* [3, 10, 12, 25, 28, 40]. Moreover, a range of studies demonstrated seropositivity among the blood donors and the control populations without a specific history of a tick bite [32]. Another report described the highest seropositivity among persons who denied having tick bites [25]. A study investigating the risk of acquiring a tick-borne pathogen after a tick bite failed to identify a significant difference between the group of persons bitten by ticks infected with *A*. *phagocytophilum* and the group bitten by uninfected ticks [40]. The possible explanations for this oversight could be that the stage of feeding ticks as nymphs and larvae may not be detectable because of their small size or that the capacity of ticks to modulate host immune and inflammatory responses may also decrease the chance of detection [12, 28]. Further, several persons from the blood donor group that were questioned about previous contact with ticks were not familiar with these parasites. Therefore, *A*. *phagocytophilum* infection should not be ruled out in the absence of self-reported previous tick exposure [4].

Most of the epidemiological surveys about *A*. *phagocytophilum* have used only the indirect IFA or the enzyme linked immunosorbent assay (ELISA). Either technique used alone with the standard cutoffs may overestimate the prevalence of antibodies [28, 40]. The World Health Organization guidelines set the cutoff at the 98^th^ percentile i.e., at 1:128, to fulfil the requirements for seroepidemiological studies. This cutoff should reduce the overestimation of the seroprevalence and therefore provide reliable information with regard to previous infections [15, 18]. The overestimation of the seroreactivity with IFA testing might be due to false-positive results secondary to potential cross-reactions [40]. These results can be observed with several other vector-borne pathogens including tick-borne encephalitis virus (6.7%), *Rickettsia conorii* (8%), *Coxiella burnetii* (10%), *Borrelia burgdorferi* (16.7%) and *Bartonella quintana* (70%) [5]. The Epstein–Barr virus infection, autoimmune disorders and *Ehrlichia* species may also induce cross-reactivity [3, 15, 33, 40]. However, two studies have failed to demonstrate an increased reactivity to *A*. *phagocytophilum* in samples that were seropositive to Epstein-Barr virus, cytomegalovirus, parvovirus B19, *Toxoplasma gondii*, *Borrelia burgdorferi* sensu lato, *Coxiella burnettii*, *Rickettsia conorii* and *E*. *chaffeensis* [15, 21]. Moreover, IFA based on HL60-cells infected with a human isolate of *A*. *phagocytophilum* are considered to be both sensitive [15] and highly specific for the investigation of seroreactivity [5]. According to the manufacturer, the specificity of this test reaches 100%, and the sensitivity depends on the period between the moment of sampling and the begining of the clinical signs, which ranges from 66.7% to 100%.

Clinical data were not recorded in our study; thus it is unknown whether the subjects who were seropositive to *A*. *phagocytophilum* experienced any clinical signs before the date of sampling. Although one previous report has found a positive association between fever in the last two years and a high seroprevalence of *A*. *phagocytophilum* [3], all or almost all seropositive persons denied any clinical symptoms of HGA in several epidemiological surveys especially in Europe [18, 25], suggesting that a high proportion of the infections could be subclinical [31]. Other possible reasons for the discrepancy between a high seroprevalence and a low incidence of the disease include underdiagnosis or misdiagnosis due to the unawareness of physicians, the circulation of variants that are non-pathogenic for humans, which may cause only transient infections without the relevant clinical signs and the potential serologic cross-reactivity with other bacteria [32, 40]. Despite a low incidence rate and because the severity of the disease is closely linked to delayed diagnosis and treatment, some authors have emphasised the importance of clinicians awareness to promptly diagnose this infection especially in high-risk areas and even in persons without a self-reported history of a tick bite [28]. At least, *A*. *phagocytophilum* should be considered in the differencial diagnosis of flu-like syndromes, febrile patients especially from high-risk areas, febrile illness of unknown aetiology or in those who are not responding to beta-lactam antibiotics or macrolides [16, 20, 21]. Only one serum sample was performed for each participant; samples were not paired and IgM were not measured. Therefore, it was not possible to estimate the incidence of seroconversion and evaluate a potential acute exposure [28]. IgM antibodies are detectable during the first 40 days after infection and IgG seroconversion occurs approximately 20–40 days after the onset of symptoms and persists for several months to years postinfection. Therefore, with a single positive IgG titer, it is not possible to distinguish between current and past exposure to *A*. *phagocytophilum* [35]. In addition, serological testing close to the onset of symptoms is usually negative [4, 6, 32]. However, IgM testing is reported to be less sensitive than IgG detection, even during early stages of infection.^4^ Sampling took place only in two cities of Morocco and subjects’ deployment histories were unavailable in the dog handler group. Therefore, no valuable data were available on human exposure in other regions of the country or the distribution or the presence of specific foci within some regions. A more comprehensive and representative study should be conducted to better estimate the prevalence of this bacterium in Morocco.

## Conclusion

To the author’s knowledge, this study is the first evidence of human exposure to *A*. *phagocytophilum* or to an antigenically similar bacterium in Morocco. The very high prevalence rate found in both high-risk populations and blood donors indicated the necessity for large-scale serologic surveys to better estimate the prevalence of this bacterium in Morocco. We hope that this study can serve as an indicator to Moroccan physicians that *A*. *phagocytophilum* infection is present and that this will help raise awareness of the potential occurrence of TBDs. Further studies especially those based on the isolation of the causative agent from patients with clinical signs compatible with HGA are warranted to clearly confirm the presence of the bacterium and to assess its role in causing disease in Morocco. Investigations of the epidemiology and the ecology of the bacterium in Morocco are also needed.
